# Quercetin Improves Postischemic Recovery of Heart Function in Doxorubicin-Treated Rats and Prevents Doxorubicin-Induced Matrix Metalloproteinase-2 Activation and Apoptosis Induction

**DOI:** 10.3390/ijms16048168

**Published:** 2015-04-13

**Authors:** Monika Barteková, Petra Šimončíková, Mária Fogarassyová, Monika Ivanová, Ľudmila Okruhlicová, Narcisa Tribulová, Ima Dovinová, Miroslav Barančík

**Affiliations:** 1 Institute for Heart Research, Slovak Academy of Sciences, Bratislava 84005, Slovakia; E-Mails: Monika.Bartekova@savba.sk (M.B.); Petra.Simoncikova@savba.sk (P.S.); maria.fogarassyova@azet.sk (M.F.); Monika.Strniskova@savba.sk (M.I.); Ludmila.Okruhlicova@savba.sk (L.O.); Narcisa.Tribulova@savba.sk (N.T.); 2 Institute of Normal and Pathological Physiology, Slovak Academy of Sciences, Bratislava 84005, Slovakia; E-Mail: Ima.Dovinova@savba.sk

**Keywords:** quercetin, doxorubicin, heart, ischemic tolerance, matrix metalloproteinases, cell signaling

## Abstract

Quercetin (QCT) is flavonoid that possesses various biological functions including anti-oxidative and radical-scavenging activities. Moreover, QCT exerts some preventive actions in treatment of cardiovascular diseases. The aim of present study was to explore effects of prolonged administration of QCT on changes induced by repeated application of doxorubicin (DOX) in rat hearts. We focused on the ultrastructure of myocardium, matrix metalloproteinases (MMPs), biometric parameters, and apoptosis induction. Our aim was also to examine effects of QCT on ischemic tolerance in hearts exposed to chronic effects of DOX, and to determine possible mechanisms underlying effects of QCT. Our results showed that QCT prevented several negative chronic effects of DOX: (I) reversed DOX-induced blood pressure increase; (II) mediated improvement of deleterious effects of DOX on ultrastructure of left ventricle; (III) prevented DOX-induced effects on tissue MMP-2 activation; and (iv) reversed effects of DOX on apoptosis induction and superoxide dismutase inhibition. Moreover, we showed that rat hearts exposed to effects of QCT were more resistant to ischemia/reperfusion injury. Effects of QCT on modulation of ischemic tolerance were linked to Akt kinase activation and connexin-43 up-regulation. Taken together, these results demonstrate that prolonged treatment with QCT prevented negative chronic effects of DOX on blood pressure, cellular damage, MMP-2 activation, and apoptosis induction. Moreover, QCT influenced myocardial responses to acute ischemic stress. These facts bring new insights into mechanisms of QCT action on rat hearts exposed to the chronic effects of DOX.

## 1. Introduction

Quercetin (QCT; 3,5,7,3',4'-pentahydroxyflavone) is a polyphenolic compound present in various foods including vegetables, fruit and wine [1]. This flavonoid possesses various biological functions including anti-oxidative, anti-inflammatory, anti-coagulation, and oxygen radical-scavenging activities [2,3,4]. Recently, QCT has been found to have certain preventive actions in the treatment of cardiovascular diseases [5,6,7,8,9,10,11]. Animal studies demonstrated that QCT exerts vasodilating and blood pressure-lowering effects in spontaneous hypertensive rats [5,6] and in rats fed a high-fat high sucrose diet [7]. An acute application of QCT before ischemia or during reperfusion has been found to protect the myocardium from ischemia/reperfusion injury [8,9]. In another study, a significant reduction of the myocardial infarct size in both normal and diabetic animals by QCT has been reported [10]. This modulation of ischemic tolerance in diabetic hearts suggests that QCT can influence ischemic tolerance in hearts affected by another prolonged pathological stress stimuli. Quercetin has also been found to have beneficial effects in combating cadmium-induced oxidative cardiotoxicity and dyslipidemia in rats [11].

An application of anthracycline doxorubicin (DOX), a known stress factor, can induce chronic pathological changes in myocardium. DOX is used in treatment of various cancers such as solid tumours, leukaemia, lymphomas and soft tissue sarcoma [12], however, its clinical utility has been hampered by its adverse cardiac effects. The mechanisms of its cardiotoxicity may be multifactorial [13], including the impairment of mitochondrial energetics by increase in the mitochondrial calcium and reactive oxygen species (ROS) leading to oxidative stress, cell necrosis and induction of pro-apoptotic signaling pathways [12,14]. An application of DOX was also found to influence sensitivity of hearts to ischemic injury. A recent study demonstrated that acute administration of DOX upon induction of ischemia significantly increased the infarct size [15]. On the other hand, a prolonged exposure of rats to DOX induced adaptive responses of myocardium associated with modulation of myocardial resistance to acute ischemic insult [16].

The aim of this study was to explore the effects of prolonged administration of quercetin on the changes induced by repeated application of doxorubicin in rat hearts. We focused on changes in the ultrastructure of myocardium, matrix metalloproteinases, biometric parameters, and apoptosis induction. Furthermore, we investigated the effects of QCT on ischemic tolerance in rat hearts exposed to the chronic effects of DOX and we also aimed to assess the molecular mechanisms underlying the effects of QCT.

## 2. Results

### 2.1. Effects of Quercetin on Biometric Parameters

The application of QCT alone did not influence registered parameters significantly. There was significant decrease of body weight and weight gain of rats in the DOX-treated group in comparison to control saline-treated animals (Table 1). Application of QCT further potentiated the DOX-induced effects on modulation of BW and weight gain; however, the changes (DOX–QCT *vs.* DOX) were not statistically significant. The application of DOX or QCT alone did not influence the weight of the whole heart and weight of the left ventricle in comparison to control conditions, and comparisons between DOX and DOX–QCT groups also did not show statistically significant changes. Eight weeks after the end of the DOX treatment, the systolic blood pressure (SBP) and heart rate were significantly increased in comparison with control animals. The treatment with QCT attenuated the DOX-induced effects and reversed the blood pressure and heart rate increase in DOX-treated rats (Table 1).

**Table 1:** Effects of quercetin on biometric parameters in normal and doxorubicin-treated rats. BW, body weight; HW, heart weight; LV, left ventricle; SBP, systolic blood pressure; HR, heart rate; C, control rats; QCT, quercetin-treated rats; DOX, doxorubicin-treated rats; DOX–QCT, rats treated with both doxorubicin and quercetin. Data are presented as the mean ± SEM (*n =* 12 per group). Statistical significance was revealed by one way ANOVA with Bonferroni *post-hoc* test and statistical differences were always determined among groups C and DOX (or QCT) (**a**) as well as DOX and DOX–QCT (**b**), ^a^ *p <* 0.05 *vs.* C, ^b^ *p <* 0.05 *vs.* DOX. Statistically significant changes are in Table marked in bold.

Parameter	C	QCT	DOX	DOX–QCT
BW (g)	432.7 ± 13.3	428.6 ± 21.3	**384.6** ± **16.8 ^a^**	355.4 ± 17.1
Weight gain (g)	154.8 ± 11.9	153.5 ± 14.4	**107.2** ± **13.2 ^a^**	89.3 ± 16.3
HW (g)	1.43 ± 0.09	1.37 ± 0.12	1.26 ± 0.07	1.11 ± 0.06
HW/BW	3.325 ± 0.196	3.162 ± 0.211	3.282 ± 0.176	3.170 ± 0.174
LV (mg)	870.6 ± 51.3	841.7 ± 86.3	822.7 ± 39.5	740.8 ± 34.0
SBP (mm·Hg)	113 ± 4	120 ± 3	**137** ± **4 ^a^**	**118** ± **5 ^b^**
HR (beats/min)	369 ± 5	376 ± 16	**392** ± **6 ^a^**	**368** ± **9 ^b^**

### 2.2. Electron Microscopic Analysis of Quercetin Effects on Ultrastructural Changes Induced by Doxorubicin

**Figure 1:**
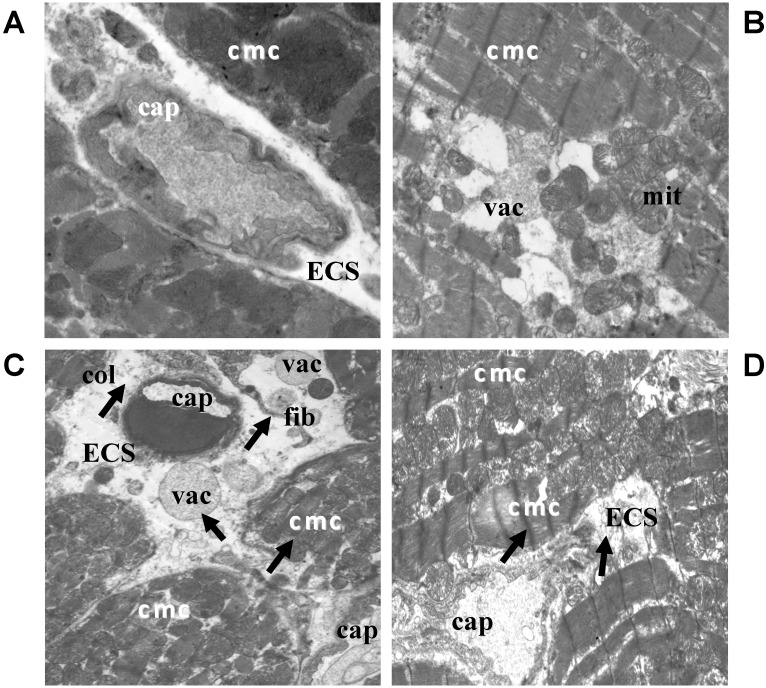
Electron microscopic images showing qualitative changes in ultrastructure of the left ventricle of rat hearts. (**A**) Electron micrograph of control rat heart showing normal architecture of cardiomyocytes and without changes in ECS; (**B**) Electron micrograph of the myocardium after treatment with QCT; (**C**) Electron micrograph of the myocardium affected with DOX demonstrating subcellular alterations of cardiomyocytes and extracellular space (arrows); (**D**) Ultrastructure of the myocardium of rats treated with both QCT and DOX. Arrows indicate improvement of some deleterious subcellular alterations induced by DOX. ECS: extracellular space; cmc: cardiomyocytes; cap: capillary; col: collagen; mit: mitochondria; vac: vacuole; fib: fibroblast. (**A**) original magnification ×8000; (**B**) original magnification ×8000; (**C**) original magnification ×6000; (**D**) original magnification ×6000.

An electron microscopic examination of the hearts of control rats showed intact ultrastructure of the myocytes, without significant abnormalities in the extracellular space (Figure 1A). The treatment of rats with QCT did not have deleterious effects on ultrastructure of the tissue of left ventricle (Figure 1B). On the other hand, the application of DOX resulted in more serious heterogeneous subcellular abnormalities of cardiomyocytes as well as extracellular space (Figure 1C). The latter was manifested by an increased density of extracellular matrix proteins, the accumulation of perivascular collagen, the presence of large and/or small vacuoles and fibroblasts with their long projections. Electron microscopy of the left ventricle of the rats exposed to the effects of both DOX and QCT (Figure 1D) showed that application of QCT mediated improvement of several deleterious subcellular alterations (of extracellular matrix) induced by DOX.

### 2.3. Quercetin Treatment Modulates the Doxorubicin-Induced Effects on Matrix Metalloproteinase-2

**Figure 2:**
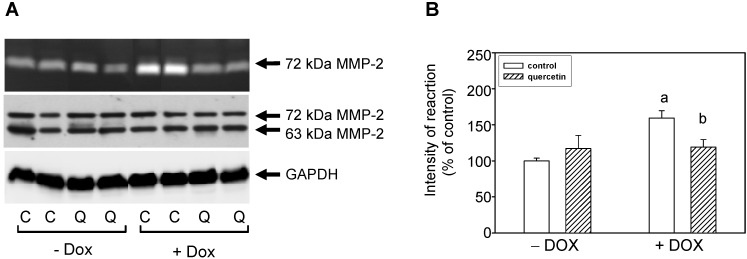
Effect of QCT and DOX treatment on tissue matrix metalloproteinase-2. (**A**) At the top is a record showing the activities of MMP-2 analyzed using gelatin zymography; in the middle, a western blot record showing MMP-2 protein levels analyzed using a specific antibody that reacts with both the 72 and 63 kDa forms of MMP-2, and at the bottom is documented the protein loading using GAPDH; (**B**) Quantitative analysis of the tissue 72-kDa MMP-2 activities. Data are expressed as a percentage of value for corresponding control. Each bar represents mean ± S.E.M. of seven independent tissue samples per group. Statistical significance is revealed by Student’s unpaired *t*-test, ^a^ *p <* 0.05 *vs.* control saline-treated (−DOX) rats; ^b^ *p <* 0.05 *vs.* DOX-treated (+DOX) rats.

The MMPs activities in heart tissue as well as in blood plasma samples were analyzed by zymography using gelatin as a substrate. The positions of 63- and 72-kDa forms of MMP-2 were identified using corresponding positive controls. In tissue of the left ventricle treatment with DOX induced up-regulation of 72-kDa MMP-2 activities but the application of QCT prevented these DOX-induced effects on tissue MMP-2 activation (Figure 2A,B). The observed effects of QCT and DOX on MMP-2 activities were not associated with a modulation of the protein levels of this enzyme. 

**Figure 3:**
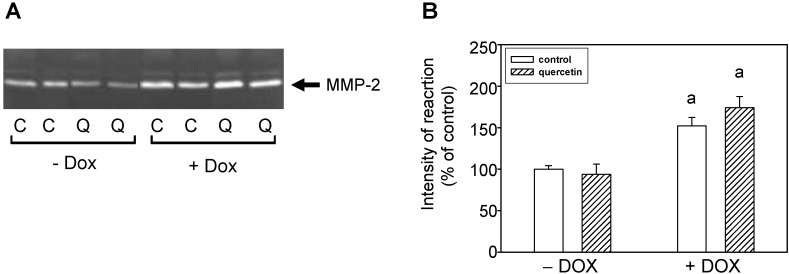
Effects of QCT and DOX treatment on plasma MMP-2 activities. (**A**) Zymogram showing the activities of circulating plasma MMP-2 analyzed using gelatin zymography; (**B**) Quantitative analysis of plasma MMP-2 activities. Data are expressed as a percentage of value for corresponding control. Each bar represents mean ± S.E.M. of seven independent plasma samples per group. Statistical significance is revealed by Student’s unpaired *t*-test, ^a^ *p <* 0.05 *vs.* control saline-treated (−DOX) rats.

By zymographic analysis of blood plasma samples we identified using positive controls the activities of 63- and 72-kDa MMP-2. We found significantly increased activities of 72-kDa MMP-2 in plasma of rats exposed to the prolonged effects of DOX (Figure 3). The observed increase in 72-kDa MMP-2 activities after DOX treatment in plasma correlated with increase of MMP-2 activities in the left ventricle. However, treatment with QCT failed to prevent the DOX-induced effects on activation of circulating plasma MMP-2 (Figure 3).

### 2.4. Quercetin Prevents the Negative Effects of Doxorubicin on Apoptosis Induction and Superoxide Dismutase Inhibition

**Figure 4:**
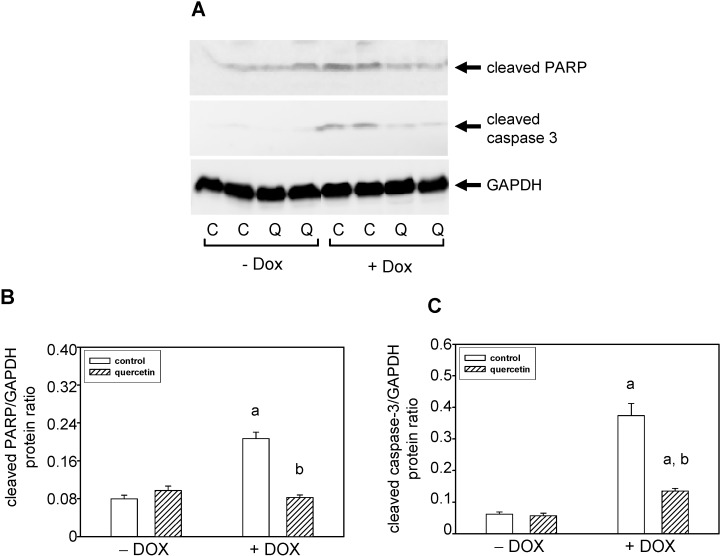
Effect of QCT and DOX on markers of apoptosis induction in the left ventricle. (**A**) The protein levels of cleaved caspase-3 and cleaved PARP were determined by western blot analysis using specific antibodies. The protein loading is documented using GAPDH; (**B**) Quantification of cleaved caspase-3 content normalized to the GAPDH protein levels; (**C**) Quantification of cleaved PARP content normalized to the GAPDH protein levels. Data were obtained from western blot records and each bar represents mean ± S.E.M. of seven tissue samples per group. Statistical significance is revealed by Student’s unpaired *t*-test, ^a^ *p <* 0.05 *vs.* control saline-treated (−DOX) rats; ^b^ *p <* 0.05 *vs.* DOX-treated (+DOX) rats.

Detection with a specific antibody documented an increased content of cleaved PARP (poly(Adenosine Diphosphate-Ribose) Polymerase) in the left ventricle of rats exposed to the prolonged effects of DOX (Figure 4A,B). Apoptosis induction by DOX was confirmed also by caspase-3 activation (Figure 4A,C). The observed data show that QCT prevented the negative effects of DOX on apoptosis induction and its application reversed the DOX-induced caspase-3 activation (Figure 4A,C) and PARP cleavage (Figure 4A,B).

**Figure 5:**
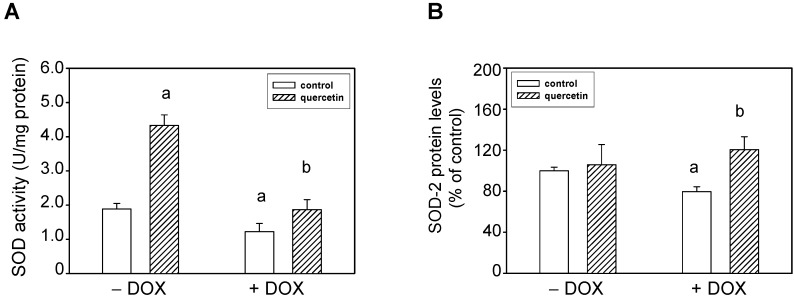
Effects of QCT and DOX treatment on superoxide dismutase (SOD) total activities and protein levels. (**A**) The SOD activities were analyzed using the SOD Assay kit in tissue samples of the left ventricle. The specific activities are expressed in units per mg of proteins and are presented as mean ± S.E.M.; (**B**) Quantitative analysis of SOD-2 protein levels. Data are expressed as a percentage of value for corresponding control. Each bar represents mean ± S.E.M. of seven independent tissue samples per group. Statistical significance is revealed by Student’s unpaired *t*-test, ^a^ *p <* 0.05 *vs.* control saline-treated (−DOX) rats; ^b^ *p <* 0.05 *vs.* DOX-treated (+DOX) rats.

The induction of apoptosis as well as activation of the non-cleaved, oxidatively activated 72-kDa form of tissue ventricular MMP-2 suggested potential alterations in activities of enzymes involved in radical (superoxide) formation. We found that the effects of DOX were associated with reduction of total superoxide dismutase (SOD) activities. QCT treatment prevented the negative effects of DOX on SOD inhibition (Figure 5A). Moreover, QCT alone induced a significant stimulation of SOD activities in control animals. The observed changes in SOD activities in DOX-treated rats were in positive correlation with protein levels of SOD-2 isoform. DOX induced down-regulation of SOD-2 protein and quercetin reversed these DOX-induced effects on SOD-2 (Figure 5B). On the other hand, QCT and DOX did not influence the protein expression of SOD-1.

### 2.5. Prolonged Application of Quercetin Improves Postischemic Recovery of Cardiac Function of Isolated Rat Hearts

Left ventricular developed pressure (LVDP, systolic minus diastolic pressure), maximal rates of pressure development +(dP/dt)_max_ and fall −(dP/dt)_max_ (as the indexes of contraction and relaxation), as well as coronary flow (CF) were used to assess cardiac function. There were no significant differences in the baseline values of all these parameters between all four groups.

**Figure 6:**
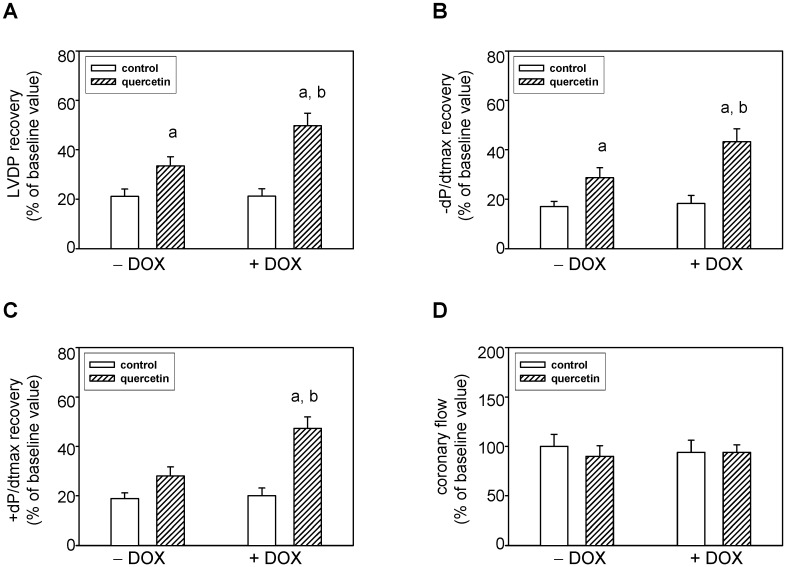
Effects of quercetin on ischemic tolerance of hearts isolated from normal and doxorubicin-treated rats. (**A**) Postischemic recovery of the left ventricular developed pressure LVDP; (**B**) Postischemic recovery of the maximal rate of pressure development +(dP/dt)_max_; (**C**) Postischemic recovery of the maximal rates of pressure fall −(dP/dt)_max_; (**D**) Postischemic recovery of the coronary flow. The recovery of parameters was determined after 30 min lasting global ischemia followed by a 40-min reperfusion. The recovery of parameters was expressed as a percentage of pre-ischemic baseline values. Each bar represents mean ± S.E.M. of eight independent measurements. ^a^ *p <* 0.05 *vs.* control saline-treated (−DOX) rats; ^b^ *p <* 0.05 *vs.* DOX-treated (+DOX) rats.

After ischemia/reperfusion, the recovery of these parameters was determined and expressed as a percentage of pre-ischemic baseline values. In the 40th minute of reperfusion, the recovery of several functional parameters of the hearts was significantly improved in both QCT-treated groups against QCT-non-treated animals (control or DOX-treated, respectively). Recovery of LVDP was significantly higher in QCT against control as well as in DOX–QCT against DOX (Figure 6A). Similarly, the maximal postischemic recovery of −(dP/dt)_max_ was significantly increased after application of QCT and that in both saline (QCT) and DOX-treated (DOX–QCT) rats (Figure 6B). A tendency of an improved recovery after the treatment with quercetin was also observed for +(dP/dt)_max_ but the changes were significant only in rats exposed to the effects of both QCT and DOX (Figure 6C). During the entire experiment there were no significant differences in the coronary flow. Figure 6D indicates the values in the 40th minute of reperfusion.

### 2.6. Cardioprotective Effects of Quercetin Are Associated with Akt Kinase Activation and Connexin-43 Up-Regulation

**Figure 7:**
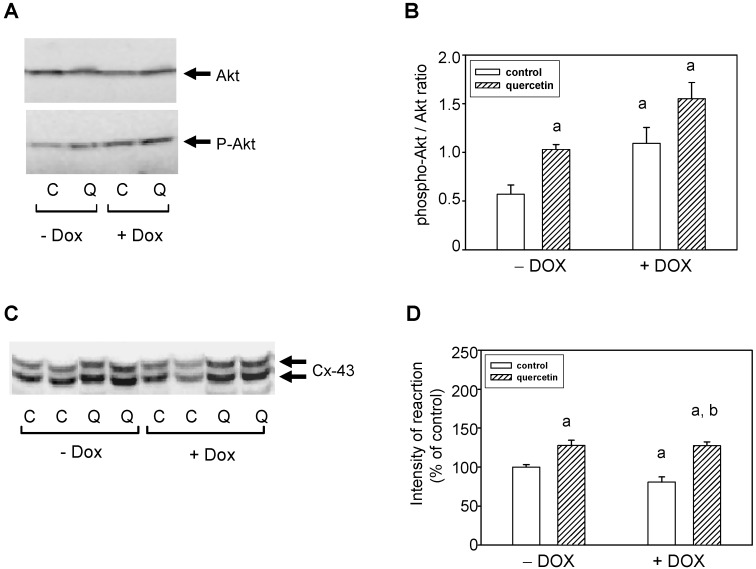
Effect of QCT and DOX on Akt kinase and connexin-43. (**A**) Western blot records showing the changes in protein levels and specific phosphorylation of Akt kinase. The changes in activation of Akt kinase were determined using an antibody which reacts with Akt kinase phosphorylated specifically on Ser473; (**B**) The quantification of Akt kinase phosphorylation (activation). Data were obtained from western blot records and are expressed as a ratio of content of phosphorylated Akt kinase to total Akt kinase. Each bar represents mean ± S.E.M. of seven tissue samples per group. Statistical significance is revealed by Student’s unpaired *t*-test, ^a^ *p <* 0.05 *vs.* control saline-treated (−DOX) rats; ^b^ *p <* 0.05 *vs.* DOX-treated (+DOX) rats; (**C**) Protein levels of Connexin-43 (Cx-43) were determined by western blot analysis using specific antibody. The upper Cx-43 band represents the expression of highly phosphorylated Cx-43, while the lower band corresponds to Cx-43 phosphorylated to a lower extent (degree); (**D**) Quantification of content of the lower phosphorylated form of Cx-43. Bars represent mean ± S.E.M. of measurements of seven independent tissue samples per group. Statistical significance is revealed by Student’s unpaired *t*-test; ^a^ *p <* 0.05 *vs.* control saline-treated (−DOX) rats; ^b^ *p <* 0.05 *vs.* DOX-treated (+DOX) rats.

To characterize the possible mechanisms involved in cardioprotective effects of QCT, we investigated the changes in Akt kinase. The obtained data showed that there are no differences in the levels of total Akt kinase between the experimental groups (Figure 7A). However, detection with a phospho-specific antibody revealed an increased phosphorylation of Akt kinase specifically on Ser473 in the left ventricle of rat hearts exposed to the effects of QCT either in saline- or doxorubicin-treated groups of rats (Figure 7A). Prolonged effects of DOX were also associated with a significantly increased specific phosphorylation of Akt kinase. From the point of the modulation of ischemic tolerance it is important that application of QCT further potentiated Akt kinase activation. The observed levels of active Ser473 phosphorylated Akt kinase (after QCT and/or QCT+DOX treatment) were also increased in relation to total Akt kinase levels (Figure 7B).

Connexin-43 (Cx-43) protein expression was analyzed using a specific antibody detecting both phosphorylated and unphosphorylated Cx-43. Doxorubicin treatment induced a reduction of Cx-43 protein, especially of low phosphorylated form of Cx-43, but quercetin treatment reversed the DOX-induced reduction of Cx-43 (Figure 7C,D). The levels of Cx-43 after application of QCT even exceed the values observed in control animals.

## 3. Discussion

The present study clearly shows that prolonged treatment with QCT possesses beneficial effects against DOX-induced changes in rat hearts as evident from lowered systolic blood pressure, decreased cellular damage, attenuated tissue MMP-2 activation, and decreased apoptosis induction. Moreover, we showed that QCT enhanced function of isolated hearts of saline- and doxorubicin-treated rats and these cardioprotective effects of QCT were realized through increased recovery of contractile function after ischemia/reperfusion.

Our data demonstrate that QCT protects by reversing DOX-induced increase of systolic blood pressure. Interestingly, the prolonged application of QCT decreased blood pressure in DOX-treated (hypertensive) animals, but not in rats with normal blood pressure (control, saline-treated). Similar antihypertensive effects of QCT were found in several animal models of increased blood pressure: this flavonoid has been shown to decrease blood pressure in spontaneously hypertensive rats [5], Dahl salt-sensitive rats [17], and rats fed with a high-fat, high-sucrose diet [7]. There exist several mechanisms potentially involved in antihypertensive effects of QCT that can include a decrease in oxidative stress as well as improved vascular function, and this is supported by antioxidant effects of QCT, and its ability to have vasodilator effects on rat arteries [18].

It has been previously demonstrated that chronic effects of anthracyclines are associated with profound changes in left ventricular morphology and function as well as with alterations in the collagen network [19]. In the present study we observed heterogeneous subcellular alterations in the left ventricle after DOX treatment and we found that application of QCT resulted in prevention of some deleterious subcellular and extracellular space changes induced by DOX. The positive effects of QCT on ultrastructure of the left ventricle were associated with prevention of MMP-2 activation. MMP-2 is an enzyme that plays an important role in processes of extracellular matrix remodeling and its activation could be related to structural disorganizations of the cardiac extracellular space. The activation of myocardial and circulating MMPs, especially MMP-2, is closely associated with the progression of the toxic effects of DOX [20] and these enzymes were also found to be markers of acute DOX-induced cardiotoxicity in mice [21]. We found modulation of activity of the 72-kDa MMP-2, the form of MMP-2 that may be oxidatively activated through conformational changes induced by radicals. This suggested potential alterations in enzyme activities of enzymes involved in radical formation. We found that the effects of DOX on 72-kDa MMP-2 activation in the left ventricle were associated with reduction of total superoxide dismutase (SOD) activities, and QCT treatment prevented the negative effects of DOX on both 72-kDa MMP-2 activation and SOD inhibition. Thus, the effects of QCT on DOX-induced changes in tissue 72-kDa MMP-2 activities can also be explained through the observed effects on recovery of SOD activities. MMP-2 is sensitive to oxidative stress and reactive oxygen species (ROS) can activate pro-MMP-2 (72-kDa MMP-2) by oxidation of the sulfide bond in the prodomain of the MMP, and peroxynitrite (a reaction product of superoxide and nitric oxide) activates pro-MMP *via* interaction with glutathione [22,23,24]. The connection between superoxide levels (SOD) and MMP-2 was also demonstrated in a study showing increased superoxide production and MMP-2 activation induced by high glucose in bovine retinal endothelial cells [25]. Addition of the MnSOD mimic MnTBAP, prevented the high glucose-induced increase of superoxide levels and ameliorated the elevation in MMP-2 activities. Moreover, both superoxide production and MMP-2 activation were prevented through an inhibitor of MMP-2 [25].

We found that treatment with QCT prevented the activation of pro-apoptotic signaling pathway induced in consequence of DOX application. QCT reduced the negative effects of DOX on apoptosis induction and reversed the DOX-induced caspase-3 activation and PARP cleavage. The observed anti-apoptotic effects of QCT in hearts of DOX-treated rats may also be related to the observed alterations in SOD activities (superoxide formation) and are in agreement with described anti-oxidative, oxygen radical-scavenging, and anti-apoptotic functions of QCT [2].

We found that the prolonged application of QCT did not have positive effects only on heart responses after chronic stress induced by DOX but mediated also the myocardial responses to additional acute ischemic stress. In the present study we showed that quercetin enhanced postischemic tolerance of isolated hearts of both doxorubicin- and saline-treated rats. The protective effects of QCT were realized through an increased recovery of contractile function after ischemia/reperfusion. The obtained data suggest that the QCT-induced protection against ischemia/reperfusion injury can be due to an observed endogenous baseline activation of Akt kinase pathway. Total levels of Akt kinase were not modified in hearts either by QCT or DOX. In contrast, a significant increase in specific phosphorylation of Akt kinase was apparent after the QCT application. As a consequence, the ratio phosphorylated/total for Akt increased in animals exposed to prolonged effects of QCT. The finding that application of QCT potentiated Akt kinase activation not only in saline but also in DOX-treated rat hearts was important, and Akt kinase activation and improvement of ischemic tolerance were maximal in animals co-treated with both QCT and DOX. Some studies showed that an acute application of QCT before ischemia (preconditioning) or during reperfusion (postconditioning) protected the myocardium from ischemia/reperfusion injury [8,9]. In our study we observed protective effects of QCT after its chronic application and the cardioprotective effects were realized several weeks after the end of the QCT application. Also interesting was the finding that in rat hearts, the cardioprotective actions of QCT were even more potentiated in rats after DOX treatment.

Application of DOX had negative effects on the ultrastructure of heart tissue, stimulation of apoptosis, and increased superoxide production but did not have negative effects on modulation of ischemic tolerance. We suggest that a possible explanation of this discrepancy and absence of negative effects of DOX on ischemic tolerance is the observed increase of P-Akt. PI3K/Akt signaling pathway was found to have an important role in the mechanisms of heart adaptation to pathological situations [26,27] and its activation was also found to play a role in infarct size-limiting mechanisms in the rat heart [28] and by prevention of injury after transient cardiac ischemia *in vivo* [29].

Several studies documented that mechanisms of the increased myocardial tolerance against injury, induced by ischemia/reperfusion, involve the PI3K/Akt/GSK-3β pathway with phosphorylation and inhibition of the glycogen synthase kinase-3β (GSK-3β) [30,31,32]. Moreover, phosphorylation of GSK-3β through PI3K/Akt pathway can lead to GSK-3β inactivation with a subsequent accumulation/activation of β-catenin [33]. Another study showed that an increase in Akt kinase activity is crucial for the increased levels of heme oxygenase-1 (HO-1) induced protection against hypoxia-induced injury. Stimulation of Akt kinase resulted in phosphorylation of GSK-3β at Ser 9. Phosphorylation at this site inhibited the activation of GSK3-beta, leading to decreased mitochondrial permeability transition pore (mPTP) opening followed by an increase in cardiomyocyte protection [34]. The Akt kinase activation observed in our study is likely to be a protective response to counteract DOX-induced negative effects on cardiac cells. The role of Akt kinase in these effects is also supported by finding that Akt kinase activation correlates well with cardioprotection (recovery of cardiac function). These two parameters were maximal in animals co-treated with both DOX and QCT.

The obtained data suggest that cardioprotective effects of QCT could also be associated with observed modulation of Gap junction-associated connexin-43 (Cx-43). This protein is critically important in many cell processes including intercellular signal transduction, control of cell proliferation, and coordinated contraction of the heart, and is suggested to play an important role also in the pathophysiology of ischemia/reperfusion injury [35,36]. We have observed that cardioprotective effects of QCT are associated with up-regulation of protein levels of Cx-43. In agreement with our data, the induction of Cx-43 was also found in cardioprotection afforded by ischemic preconditioning and pretreatment with diazoxide in isolated rat hearts [35]. We have demonstrated that exposure of rats to chronic effects of DOX suppressed expression of Cx-43 in the left ventricle and QCT treatment reversed the DOX-induced effects. An inhibition of cardiac expression of Cx43 was also demonstrated 14 days after DOX administration in mice hearts was observed [37].

## 4. Experimental Section

### 4.1. Materials

The SOD Assay kit and antibody against connexin-43 were purchased from Sigma Aldrich (St. Louis, MO, USA). Primary antibodies recognizing phosphorylated Akt kinase (Ser473), cleaved caspase-3, cleaved PARP, and peroxidase-labeled anti-rabbit or anti-mouse immunoglobulins were purchased from Cell Signaling Technology (Danvers, MA, USA). Antibodies against total Akt kinase, MMP-2, SOD-1, SOD-2, and GAPDH were obtained from Santa Cruz Biotechnology (Santa Cruz, CA, USA).

### 4.2. Experimental Model

In the study, 11-weeks old male Wistar rats were used. All animals were housed at a temperature of 22–24 °C and fed with a regular pellet diet *ad libitum*. Rats were divided into four experimental groups: control saline-treated (C), doxorubicin-treated (DOX), quercetin-treated (QCT), and doxorubicin+quercetin-treated (DOX–QCT). In the DOX groups, the rats received DOX in cumulative dose 15 mg/kg for 3 weeks. DOX was applied by seven intraperitoneal injections, every 3rd day. The animals of experimental groups without application of DOX were treated with saline. Quercetin was served on a piece of bisquit in doses of 20 mg/kg/day. The administration of QCT started on the same day as the DOX (saline) administration and continued for a further 3 weeks after the completion of the DOX treatment (total period of 6 weeks). Eight weeks after the completion of DOX or saline treatment, the animals were anaesthetized with thiopental (50 mg·kg^−1^, intraperitoneally); excised hearts were either perfused according to Langendorff and tested on I/R injury or used for separation of the ventricular tissue samples. All animal experiments were performed in accordance with the rules issued by the State Veterinary Administration of the Slovak Republic, legislation No 289/2003 and with the regulations of the Animal Research and Care Committee of Institute for Heart Research SAS—Project 1873/11-221/3, approved on 30 September 2011.

### 4.3. Systolic Blood Pressure and Heart Rate Measurement

The systolic blood pressure (SBP) and heart rate (HR) were measured by the non-invasive method of tail cuff plethysmography (PowerLab 4/30, ADInstruments, Budapest, Hungary) in all experimental groups of rats. The measurements were performed before the first DOX or saline applications and eight weeks after the end of their application.

### 4.4. Perfusion Technique and Determination of Heart Function

Rats were anaesthetized (thiopental, 50 mg/kg, i.p.) and heparinised (500 IU, i.p.) before a heart excision and perfusion. Hearts were rapidly excised, placed in ice-cold perfusion buffer, cannulated via the aorta and placed onto the Langendorff setup for perfusion at a constant perfusion pressure of 73 mm Hg and temperature of 37 °C. Perfusion solution was a modified Krebs–Henseleit buffer gassed with 95% O_2_ and 5% CO_2_ (pH 7.4) containing (in mmol/L): NaCl 118.0; KCl 3.2; MgSO_4_ 1.2; NaHCO_3_ 25.0; KH_2_PO_4_ 1.18; CaCl_2_ 2.5; glucose 7.0. This solution was filtered through a 5 µm porosity filter to remove contaminants. An epicardial electrogram was registered by means of two stainless steel electrodes attached to the apex of the heart and aortic cannula. Left ventricular pressure was measured by means of a water-filled balloon inserted into the left ventricle via the left atrium (adjusted to obtain end-diastolic pressure of 1–8 mm Hg) and connected to a pressure transducer. Left ventricular developed pressure (LVDP, systolic minus diastolic pressure), maximal rates of pressure development and fall, +(dP/dt)_max_ and −(dP/dt)_max_, as the indexes of contraction and relaxation, as well as the heart rate (calculated from EG) and coronary flow were used to assess cardiac function. Recovery of these parameters after ischemia/reperfusion was expressed as a percentage of pre-ischemic baseline values. Global ischemia was maintained for 30 min, followed by a 40 min reperfusion. Functional parameters of hearts were measured during the whole reperfusion.

### 4.5. Samples Collection

At the end of the experiment, the animals were anesthetized with thiopental (50 mg/kg, i.p.) injection, and were euthanized by thoracotomy and rapid excision of their hearts. Excised hearts were weighted and separated to the ventricles. The whole heart (HW) and left ventricular weights (LVW) were registered. Further processing of the collected left ventricular tissue samples was dependent on the following assay. The tissue left ventricular samples for biochemical analysis were immediately frozen in liquid nitrogen and stored at −75 °C until use. For the transmission electron microscopy studies, small blocks of transmural left ventricular tissues were fixed in buffered 2.5% glutaraldehyde immediately after collection.

The plasma samples were prepared from whole artery blood drawn from the chest of the rats immediately after excision of the heart. Citrate was immediately added to the collected blood (resulting concentration 0.76%), followed by centrifugation of the blood for five min at 1200× *g* to obtain the plasma. The prepared plasma samples were stored at −20 °C until further analysis.

### 4.6. Transmission Electron Microscopy

Small (1–2 mm^3^) transmural left ventricular heart tissue samples were routinely processed for electron microscopy. The samples were fixed in 2.5% glutaraldehyde in 100 mmol/L cacodylate buffer at 4 °C, washed, postfixed in 1% OsO_4_, and subsequently embedded in Epon 812. Ultrathin sections of the tissue were stained with uranyl acetate and lead citrate. The ultrastructure of the myocardial tissue was evaluated using a transmission electron microscope Tesla 500 (Tesla, Brno, Czech Republic).

### 4.7. Preparation of Tissue Protein Fractions and Western Blot Analysis

The tissue samples were resuspended in ice-cold buffer A containing (in mmol/L): 20 Tris-HCl, 250 sucrose, 1.0 EGTA, 1.0 dithiothreitol (DTT), 1.0 phenylmethylsulphonyl fluoride (PMSF) and 0.5 sodium orthovanadate (pH 7.4) and homogenized with a Teflon homogenizer. The homogenates were centrifuged at 800× *g* for 5 min at 4 °C, the pellets were discarded after centrifugation and the supernatants were centrifuged again at 16,100× *g* for 30 min. The supernatants after the second centrifugation were used for biochemical analysis and protein concentrations were estimated by the Bradford method [38].

For western blot analysis, samples containing equivalent amounts of proteins per lane were separated by SDS-polyacrylamide gel electrophoresis (SDS-PAGE). The proteins after electrophoretic separation were transferred onto nitrocellulose membranes, and after blocking of non-specific binding sites, the membranes were incubated overnight at 4 °C with the corresponding specific primary antibody. The corresponding peroxidase-labeled anti-rabbit or anti-mouse immunoglobulins were used as secondary antibodies. Peroxidase reactions were detected by the enhanced chemiluminescence (ECL) system.

### 4.8. Measurement of MMP-2 Activities by Gelatin Zymography

The activity of MMP-2 was evaluated using zymography in 10% polyacrylamide gels containing gelatin (2 mg/mL) as a substrate for MMP-2. The samples were suspended in Laemmli buffer without 2-mercaptoethanol and loaded onto gels without denaturation. After electrophoresis, the gels were washed twice for 20 min each with 50 mmol/L Tris-HCl (pH 7.4), containing 2.5% Triton X-100, and then incubated overnight at 37 °C in a substrate buffer containing 50 mmol/L Tris-HCl, 10 mmol/L CaCl_2_ and 1.25% Triton X-100, pH 7.4. After incubation, the gels were stained with 1% Coomassie Brilliant Blue G-250 and then destained with 40% methanol and 10% acetic acid. The gelatinolytic activities of the MMPs were detected as transparent bands against a dark blue background. Recombinant, active MMP-2 was used as a positive control to identify the active 63 kDa MMP-2 form. Seventy-two kDa MMP-2 was identified using fetal bovine serum containing this form of MMP.

### 4.9. Determination of Superoxide Dismutase Activity

The SOD activity was analyzed using a SOD Assay kit, which assays SOD activity by utilizing a highly water-soluble tetrazolium salt, WST-1 (2-(4-Iodophenyl)-3-(4-nitrophenyl)-5-(2,4-disulfophenyl)-2H-tetrazolium, monosodium salt), which produces a water-soluble formazan dye upon reduction with superoxide anion. The activities were determined following the manufacturer’s instructions. The changes in formazan production were analyzed for 30 min at 37 °C using a microplate reader (Thermo Scientific Multiscan FC, Vantaa, Finland). The SOD activities were calculated using a SOD standard curve and expressed as U·mg^−1^ of protein.

### 4.10. Statistical Evaluation

Statistical differences were always determined between two groups; the effects of DOX and/or QCT were compared to the control conditions (DOX or QCT groups *vs.* control C group) and QCT with DOX to the conditions when only DOX was applied (DOX–QCT *vs.* DOX group).

Statistical significance was revealed by one-way ANOVA with Bonferroni *post-hoc* test or Student’s unpaired *t*-test (Origin software). Differences were considered significant at *p <* 0.05 in all the tests.

## 5. Conclusions

Taken together, our results demonstrate that prolonged treatment with QCT prevented negative chronic effects of DOX on blood pressure, cellular damage, MMP-2 activation, and apoptosis induction in hearts. Treatment with QCT had positive effects not only on heart responses after chronic stress induced by DOX, but also mediated myocardial responses to an additional acute ischemic stress. The findings of the current study demonstrate that a prolonged application of QCT improved ischemic tolerance and that this improvement was in concert with triggering of Akt kinase activation and Cx-43 up-regulation.

## References

[1] Hertog M.G., Bueno-de-Mesquita H.B., Fehily A.M., Sweetnam P.M., Elwood P.C., Kromhout D. (1996). Fruit and vegetable consumption and cancer mortality in the Caerphilly Study. Cancer Epidemiol. Biomark. Prev..

[2] Erden Inal M., Kahraman A. (2000). The protective effect of flavonol quercetin against ultraviolet a induced oxidative stress in rats. Toxicology.

[3] Wu J., Xu X., Li Y., Kou J., Huang F., Liu B., Liu K. (2014). Quercetin, luteolin and epigallocatechin gallate alleviate TXNIP and NLRP3-mediated inflammation and apoptosis with regulation of AMPK in endothelial cells. Eur. J. Pharmacol..

[4] Yu P.X., Zhou Q.J., Zhu W.W., Wu Y.H., Wu L.C., Lin X., Chen M.H., Qiu B.T. (2013). Effects of quercetin on LPS-induced disseminated intravascular coagulation (DIC) in rabbits. Thromb. Res..

[5] Duarte J., Perez-Palencia R., Vargas F., Ocete M.A., Perez-Vizcaino F., Zarzuelo A., Tamargo J. (2001). Antihypertensive effects of the flavonoid quercetin in spontaneously hypertensive rats. Br. J. Pharmacol..

[6] Sanchez M., Galisteo M., Vera R., Villar I.C., Zarzuelo A., Tamargo J., Pérez-Vizcaíno F., Duarte J. (2006). Quercetin downregulates NADPH oxidase, increases eNOS activity and prevents endothelial dysfunction in spontaneously hypertensive rats. J. Hypertens..

[7] Yamamoto Y, Oue E. (2006). Antihypertensive effect of quercetin in rats fed with a high-fat high-sucrose diet. Biosci. Biotechnol. Biochem..

[8] Jin H.B., Yang Y.B., Song Y.L., Zhang Y.C., Li Y.R. (2012). Protective roles of quercetin in acute myocardial ischemia and reperfusion injury in rats. Mol. Biol. Rep..

[9] Bartekova M., Carnicka S., Pancza D., Ondrejcakova M., Breier A., Ravingerova T. (2010). Acute treatment with polyphenol quercetin improves postischemic recovery of isolated perfused rat hearts after global ischemia. Can. J. Physiol. Pharmacol..

[10] Annapurna A., Reddy C.S., Akondi R.B., Rao S.R. (2009). Cardioprotective actions of two bioflavonoids, quercetin and rutin, in experimental myocardial infarction in both normal and streptozotocin-induced type I diabetic rats. J. Pharmacy Pharmacol..

[11] Milton Prabu S, Muthumani M., Shagirtha K. (2013). Quercetin potentially attenuates cadmium induced oxidative stress mediated cardiotoxicity and dyslipidemia in rats. Eur. Rev. Med. Pharmacol. Sci..

[12] Minotti G., Menna P., Salvatorelli E., Cairo G., Gianni L. (2004). Anthracyclines: molecular advances and pharmacologic developments in antitumor activity and cardiotoxicity. Pharmacol. Rev..

[13] Arola O.J., Saraste A., Pulkki K., Kallajoki M., Parvinen M., Voipio-Pulkki L.M. (2000). Acute doxorubicin cardiotoxicity involves cardiomyocyte apoptosis. Cancer Res..

[14] Singal P.K., Iliskovic N. (1998). Doxorubicin-induced cardiomyopathy. N. Engl. J. Med..

[15] Gharanei M., Hussain A., Janneh O., Maddock H.L. (2013). Doxorubicin induced myocardial injury is exacerbated following ischaemic stress via opening of the mitochondrial permeability transition pore. Toxicol. Appl. Pharmacol..

[16] Simoncíkova P., Ravingerova T., Barancik M. (2008). The effect of chronic doxorubicin treatment on mitogen-activated protein kinases and heat stress proteins in rat hearts. Physiol. Res..

[17] Mackraj I., Govender T., Ramesar S. (2008). The antihypertensive effects of quercetin in a salt-sensitive model of hypertension. J. Cardiovasc. Pharmacol..

[18] Rendig S.V., Symons J.D., Longhurst J.C., Amsterdam E.A. (2001). Effects of red wine, alcohol, and quercetin on coronary resistance and conductance arteries. J. Cardiovasc. Pharmacol..

[19] Adamcová M., Potacova A., Popelova O., Sterba M., Mazurova Y., Aupperle H., Geršl V. (2010). Cardiac remodeling and MMPs on the model of chronic daunorubicin-induced cardiomyopathy in rabbits. Physiol. Res..

[20] Ivanova M., Dovinova I., Okruhlicová L., Tribulova N., Simoncíkova P., Bartekova M., Vlkovicova J., Barancik M. (2012). Chronic cardiotoxicity of doxorubicin involves activation of myocardial and circulating matrix metalloproteinases in rats. Acta Pharmacol. Sin..

[21] Bai P., Mabley J.G., Liaudet L., Virag L., Szabo C., Pacher P. (2004). Matrix metalloproteinase activation is an early event in doxorubicin-induced cardiotoxicity. Oncol. Rep..

[22] Okamoto T, Akaike T., Sawa T., Miyamoto Y., van derVliet A., Maeda H. (2001). Activation of matrix metalloproteinases by peroxynitrite-induced protein *S*-glutathiolation via disulfide *S*-oxide formation. J. Biol. Chem..

[23] Schulz R. (2007). Intracellular targets of matrix metalloproteinase-2 in cardiac disease: Rationale and therapeutic approaches. Annu. Rev. Pharmacol. Toxicol..

[24] Viappiani S., Nicolescu A.C., Holt A., Sawicki G., Crawford B.D., Leon H., van Mulligen T., Schulz R. (2009). Activation and modulation of 72 kDa matrix metalloproteinase-2 by peroxynitrite and glutathione. Biochem. Pharmacol..

[25] Kowluru R.A., Kanwar M. (2009). Oxidative stress and the development of diabetic retinopathy: Contributory role of matrix metalloproteinase-2. Free Radic. Biol. Med..

[26] Hausenloy D.J., Mocanu M.M., Yellon D.M. (2004). Cross-talk between the survival kinases during early reperfusion: Its contribution to ischemic preconditioning. Cardiovasc. Res..

[27] Strniskova M., Ravingerova T., Neckar J., Kolar F., Pastorekova S., Barancik M. (2006). Changes in the expression and/or activation of regulatory proteins in rat hearts adapted to chronic hypoxia. Gen. Physiol. Biophys..

[28] Ravingerova T., Matejikova J., Neckar J., Andelova E., Kolar F. (2007). Differential role of PI3K/Akt pathway in the infarct size limitation and antiarrhythmic protection in the rat heart. Mol. Cell. Biochem..

[29] Matsui T., Tao J., del Monte F., Lee K.H., Li L., Picard M., Force T.L., Franke T.F., Hajjar R.J., Rosenzweig A. (2001). Akt activation preserves cardiac function and prevents injury after transient cardiac ischemia *in vivo*. Circulation.

[30] Miura T., Tanno M., Sato T. (2010). Mitochondrial kinase signaling pathways in myocardial protection from ischaemia/reperfusion-induced necrosis. Cardiovasc. Res..

[31] Budas G.R., Sukhodub A., Alessi D.R., Jovanovic A. (2006). 3' Phosphoinositidedependent kinase-1 is essential for ischemic preconditioning of the myocardium. FASEB J..

[32] Yin Z., Gao H., Wang H., Li L., Di C., Luan R., Tao L. (2009). Ischemic postconditioning protects both adult and aged Sprague Dawley rat hearts from ischemia/reperfusion injury through the PI3-K/Akt and GSK-3β pathway. Clin. Exp. Pharmacol. Physiol..

[33] Ding V.W., Chen R.H., McCormick F. (2000). Differential regulation of glycogen synthase kinase 3β by insulin and Wnt signaling. J. Biol. Chem..

[34] Issan Y., Kornowski R., Aravot D., Shainberg A., Laniado-Schwartzman M., Sodhi K., Abraham N.G., Hochhauser E. (2014). Heme oxygenase-1 induction improves cardiac function following myocardial ischemia by reducing oxidative stress. PLoS ONE.

[35] Srisakuldee W., Jeyaraman M.M., Nickel B.E., Tanguy S., Jiang Z.S., Kardami E. (2009). Phosphorylation of connexin-43 at serine 262 promotes a cardiac injury-resistant state. Cardiovasc. Res..

[36] Garcia-Dorado D., Rodriguez-Sinovas A., Ruiz-Meana M. (2004). Gap junction-mediated spread of cell injury and death during myocardial ischemia-reperfusion. Cardiovasc. Res..

[37] Zhang H., Zhang A., Guo C., Shi C., Zhang Y., Liu Q., Sparatore A, Wang C. (2011). *S*-diclofenac protects against doxorubicin-induced cardiomyopathy in mice via ameliorating cardiac gap junction remodeling. PLoS ONE.

[38] Bradford M. (1976). A rapid and sensitive method for the quantitation of microgram quanties of protein utilizing the principle of protein-dye-binding. Anal. Biochem..

